# Incidence and Implication of Coronary Artery Calcium on Non-gated Chest Computed Tomography Scans: A Large Observational Cohort

**DOI:** 10.7759/cureus.6218

**Published:** 2019-11-22

**Authors:** Charles Haller, Anthony Vandehei, Raymond Fisher, Joshua Boster, Brian Shipley, Christopher Kaatz, Jaclyn Harris, Satoshi R Shin, Lisa Townsend, Jessica Rouse, Sarah Davis, James Aden, Dustin Thomas

**Affiliations:** 1 Cardiology, Brooke Army Medical Center, Fort Sam Houston, USA; 2 Internal Medicine, Brooke Army Medical Center, Fort Sam Houston, USA; 3 Internal Medicine, Darnall Army Medical Center, Fort Hood, USA; 4 Statistics, Brooke Army Medical Center, Fort Sam Houston, USA

**Keywords:** coronary artery calcium, computed tomography

## Abstract

Introduction

Coronary artery calcification (CAC) scoring is typically performed utilizing non-contrast, electrocardiogram- (ECG) gated CT and offers an estimation of cardiovascular (CV) prognosis and risk stratification beyond previously established cardiac risk factors. Coronary calcification can also be assessed during non-gated chest CT, which is significant given the recent recommendations for lung cancer screening by low-dose CT.

Methods

We retrospectively reviewed 4,953 non-contrast chest CT scans in a single, closed referral tertiary military treatment facility over an 18-month period. Baseline CV outcomes to include myocardial infarction (MI), cerebral vascular accidents (CVA), revascularization with percutaneous coronary intervention (PCI) or coronary artery bypass grafting (CABG), death, or a composite of all major adverse cardiac events (MACE), and baseline CV risk factors were abstracted from an electronic medical record (EMR) review.

Results

CAC was seen in 3,119 (63%) patients while 1,834 (27%) were without CAC. All traditional CV risk factors were more commonly observed in patients with CAC. Unadjusted odds of composite MACE, death, MI, coronary revascularization, and CVA between presence and absence of CAC were as follows: 3.55 [95% confidence interval (CI): 2.60-4.86, p: <0.0001]; 2.98 (95% CI: 2.02-4.40, p: <0.0001); 24.42 (95% CI: 3.36-177.6, p: <0.0001); 5.64 (95% CI: 2.58-12.32, p: <0.0001); and 2.32 (95% CI: 1.19-4.50, p: 0.0104), respectively. However, after adjusting for baseline risk factors, CAC on non-gated CT was associated only with an increased observed rate of MI (aOR: 38.1, 95% CI: 4.57-318.2, p: <0.0001) and revascularization (aOR: 5.58, 95% CI: 2.22-14.0, p; 0.0003).

Conclusions

Findings of CAC on non-gated chest CT may help to recognize patients who are at increased risk of MI and revascularization. Given the expected increase in chest CT utilization among former smokers for lung cancer screening, observed CAC should be reported to ordering providers in order to identify patients at increased risk of these important outcomes.

## Introduction

Coronary artery calcium (CAC) is a well-validated risk marker of coronary artery disease (CAD). It adds significant predictive power over traditional risk assessment factors and tools such as carotid media intimal thickness [1-4]. A plethora of data supports a strong relationship between the presence and extent of coronary calcification and clinical outcomes. This is demonstrated among multiple patient cohorts with various risk factors and ethnicities [5].

CAC scoring is traditionally performed utilizing electrocardiogram (ECG) gating with standard reconstruction and acquisition parameters (2-3-mm slice thickness and 120 peak kV) as classically described by Agatston et al. [6]. Published data suggests a good correlation among CAC scores between non-gated chest CT scans and formal CAC testing [7-9]. Furthermore, multiple small investigations have reported an increased incidence of adverse cardiovascular (CV) events and death in patients with qualitative coronary calcification on non-contrast, non-gated CT chest scans [10-14]. This is important in light of the recent recommendation from the US Preventive Services Task Force (USPSTF), released in 2014, which recommended low-dose CT screening for high-risk current and former smokers [15]. This recommendation carries the possibility of screening an additional 7-10 million patients who are at risk for CV events, with several societies, including the Society of Cardiovascular Computed Tomography (SCCT), recognizing the potential benefit to interpretation and reporting of CAC on these scans [16].

The primary objective of this analysis is to further analyze and inform on the risk for major CV events among patients with and without CAC on non-gated chest CT and assess whether non-gated chest-CT-derived CAC is an independent risk factor for CV outcomes.

## Materials and methods

Study population 

This study involves a single-center, retrospective observational cohort of patients who underwent a non-contrast, non-gated chest CT scan at Brooke Army Medical Center, Fort Sam Houston, TX between January 1, 2011 and June 30, 2012. The local picture archiving and communication system (PACS) was queried for all scans named “CT CHEST W/O CONTRAST” between the dates listed. This query was then refined by the inclusion criteria of a specified patient-age range at the time of scan: between 18 and 80 years. 

Coronary artery calcium assessment on non-gated chest CT 

Each scan was reviewed by cardiology staff credentialed in CAC interpretation and cardiology fellows trained in CAC interpretation. Each study was reviewed and marked in a binary fashion as either having or not having coronary calcification, a strictly qualitative assessment. Semi-quantitative analysis of CAC, such as severity, was not reported.

Demographics 

The identified study population was then queried in the local electronic medical record (EMR) for the presence of CV risk factors and prior use of or the initiation of lipid-lowering medications or aspirin around the time of the imaging study. CV risk factors identified included diabetes mellitus type 2 (DM2), hypertension (HTN) (defined as diagnosis of hypertension by a healthcare provider or ≥2 readings of ≥140/90 mmHg at outpatient visits), hyperlipidemia (HLP) (defined as diagnosis of hyperlipidemia by a healthcare provider or LDL of ≥190 mg/dL), active or recent smoker (recent smoker defined as any instance of smoking within six months of index scan), or known CAD (defined as any atherosclerotic plaque on coronary computed tomography angiography (CCTA) or invasive coronary angiography (ICA), prior percutaneous coronary intervention (PCI), or prior coronary artery bypass graft (CABG)).

Outcomes 

Major adverse cardiovascular events (MACE) defined as death, myocardial infarction (MI), cerebrovascular accident (CVA), or revascularization (either PCI or CABG) were abstracted. The instances of death were determined by utilizing the social security death index (SSDI). The remaining MACE outcomes were determined by the EMR chart review. Preliminary data was presented as a conference poster (Conference poster: Fisher R, Haller C, Shipley B, et al.:* *Incidence and Implication of Coronary Artery Calcium (CAC) on Non-gated Chest CT Scans: A Large Observational Cohort. The Journal of Cardiovascular Computed Tomography. 14th Annual Scientific Meeting of the Society of Cardiovascular Computed Tomography; July 13, 2019).

Statistical analysis

Continuous variables were analyzed utilizing 2-sided chi-squared testing. The comparison of means was performed using analysis of variance (ANOVA). The comparison of non-normally distributed continuous variables was reported as medians with inter-quartile ranges and analyzed using the Mann-Whitney test. The Kaplan-Meier analysis was performed for event-free survival.

## Results

CAC was diagnosed in 3,119 (63%) of the patients overall. All traditional CV risk factors (Table 1) were more commonly observed in patients with CAC compared with those without detectable CAC on non-gated chest CT (p: <0.0001).

**Table 1:** Comparison of demographics and pharmaceutical therapy for patients with coronary artery calcium and those without coronary artery calcium at the time of non-gated CT CAC: coronary artery calcium; DM2; diabetes mellitus type 2; HTN: hypertension; HLP: hyperlipidemia; CAD: coronary artery disease; CT: computed tomography; n: number *standard deviation

	CAC (n = 3,119)	No CAC (n = 1,834)	P-value
Age, years	71 (±11)*	48 (±17)*	<0.0001
Male gender	1,793 (57.5%)	891 (48.6%)	<0.0001
Smoker	1,346 (43.2%)	493 (26.9%)	<0.0001
DM2	938 (30.1%)	252 (13.7%)	<0.0001
HTN	2,578 (82.7%)	839 (45.7%)	<0.0001
HLP	2,321 (74.4%)	704 (38.4%)	<0.0001
Known CAD	1,148 (36.8%)	178 (9.7%)	<0.0001
Pre-CT statin	1960 (62.8%)	440 (24.0%)	<0.0001
Pre-CT nonstatin lipid-lowering therapy	451 (14.5%)	106 (5.8%)	<0.0001
Pre-CT Aspirin	1,827 (58.6%)	433 (23.6%)	<0.0001

CAC on non-gated CT was associated with an increased incidence of composite MACE [odds ratio (OR): 3.55, 95% CI: 2.60-4.86, p: <0.0001), death (OR: 2.98, 95% CI: 2.02-4.40, p: <0.0001), MI (OR: 24.42, 95% CI: 3.36-177.6, p: <0.0001), coronary revascularization (OR: 5.64, 95% CI: 2.58-12.32, p: <0.0001), and CVA (OR: 2.32, 95% CI: 1.19-4.50, p: 0.0104 (Table 2). However, after adjusting for baseline risk factors, CAC on non-gated CT was associated only with an increased observed rate of MI [adjuste odds ration (aOR): 38.1, 95% CI: 4.57-318.2, p: <0.0001] and revascularization (aOR: 5.58, 95% CI: 2.22-14.0, p: 0.0003). 

**Table 2:** The odds of major adverse cardiovascular event with qualitative coronary artery calcium by non-gated chest computed tomography with adjustment for traditional cardiovascular risk factors OR: odds ratio; CI: confidence interval; MACE: major adverse cardiovascular event; MI: myocardial infarction; CVA: cerebrovascular accident

Outcome	Unadjusted OR	95% CI	P-value	Adjusted OR	95% CI	P-value
Composite MACE	3.55	2.60-4.86	<0.0001	1.39	0.96-2.02	0.082
Death	2.98	2.02-4.40	<0.0001	0.94	0.59-1.49	0.779
MI	24.42	3.36-177.6	<0.0001	38.1	4.57-318.2	<0.0001
Revascularization	5.64	2.58-12.32	<0.0001	5.58	2.22-14.0	0.0003
CVA	2.32	1.19-4.50	0.0104	0.72	0.34-1.52	0.3871

## Discussion

The presence of CAC on non-gated chest CT scans correlated well with traditional cardiac risk factors, including age, male gender, smoking, DM2, HTN, and HLP. These patients with CAC were also more likely to experience MACE, including composite MACE, death, MI, coronary revascularization, and CVA by unadjusted OR. Significantly increased incidence of MI and coronary revascularization remained even after adjusting for baseline risk factors. These findings emphasize the importance of the use of non-gated chest CT scans for the identification of CAC and reinforce the correlation of visual-qualitative CAC with MACE.

Identification of CAC by non-gated chest CT scans may represent an additional means to risk-stratify patients at risk for MACE. Of patients with visual-qualitative CAC by these studies, only 36.8% had known CAD. Therefore, 63.2% of patients with coronary atherosclerosis had not been identified at the time of the scan. These patients perhaps could benefit from cardio-protective pharmacotherapy and lifestyle modification. Approximately 40% of patients with visual-qualitative CAC by non-contrast chest CT were not on statin, and nearly half of the patients were not on aspirin at the time of the exam. 

Although CAC scoring is traditionally performed utilizing ECG gating with standard reconstruction and acquisition parameters, recent publications suggest a good correlation among CAC scores from non-gated chest CT scans and formal CAC testing [7-9]. Several investigations have also demonstrated an increased incidence of MACE associated with CAC, qualitative and quantitative by non-gated chest CT scans [10-14]. Hughes-Austin et al. found a significant increase in the incidence of all-cause mortality for patients with CAC by 6-mm non-gated chest CT scans and demonstrated a correlation with traditional 3-mm ECG-gated CT scans, which remained even after adjusting for traditional CV risk factors [11]. Xie et al. reported a correlation between death and MACE with quantitative and qualitative CAC by non-gated CT in a meta-analysis of five studies [10]. Of note, although trends toward increased death and MACE were noted with CAC, several studies failed to show the significance for these outcomes in subjects with lower quantitative CAC, particularly when adjusted for traditional cardiac risk factors.

This study’s findings corroborate the role of qualitative CAC as a predictor of MACE and is strengthened by both a large patient population and subgroup analysis of events with adjustment for traditional cardiac risk factors over a long follow-up period. With the rise in non-gated chest CT scans associated with the USPSTF lung cancer screening recommendations, standardization of reporting CAC for these scans may identify patients at risk for MACE. This provides the opportunity for preventative measures by the ordering provider, such as recommendations for lifestyle modification and prescription of cardio-protective pharmacotherapy to be instituted. 

This trial was limited to a strictly visual-qualitative assessment of the presence or absence of CAC. Applying quantitative and semi-quantitative analysis to these non-gated chest CT scans, such as the non-gated Agatston score and other ordinal scoring systems, may further enhance the predictive value for future MACE to levels seen in traditional gated studies. Further limitations include an absence of assessment for variation between investigators and the role such variation may have had on odds, and a failure to compare findings to ECG-gated cardiac CT scans.

## Conclusions

Findings of CAC on non-gated CT may help identify patients who are at increased risk of MI and revascularization. Given the expected increase in chest CT utilization among former smokers for lung cancer screening, observed CAC should be reported to ordering providers in order to identify patients at increased risk of these important outcomes.
